# Analysis of the current state and influencing factors of loneliness among Chinese children aged 8–18 years

**DOI:** 10.3389/fpubh.2026.1798442

**Published:** 2026-05-18

**Authors:** Lijie Yang, Jing Zhang, Yuehang Sun, Ming Yang, Jiming Yang, Yang Yu, Zhongmei Liu, Ximu Sun, Han Zhou, Xiaolin Xu

**Affiliations:** 1 Department of Pharmacy, The Sixth Affiliated Hospital of Harbin Medical University, Harbin, China; 2 School of Pharmacy, Xuzhou Medical University, Xuzhou, China; 3 Department of Pharmacy, Beijing Children's Hospital, National Center for Children's Health, Capital Medical University, Beijing, China

**Keywords:** children, intimate partner violence, loneliness, media contact, perceived stress

## Abstract

**Objective:**

The phenomenon of childhood loneliness extends beyond its implications for psychological development, potentially exerting significant effects on mental health and social adaptation. This study investigates the impact of intimate partner violence (IPV), perceived stress, and media exposure on childhood loneliness, offering empirical evidence to enhance understanding and inform strategies to address this issue.

**Methods:**

This study adopted a cross-sectional survey design and employed stratified and quota sampling techniques to select children’s hospitals across 18 provinces and municipalities in mainland China. Participants were assessed for intimate partner violence using the IPV scale, perceived stress using the Perceived Stress Scale-4 (PSS-4), media exposure using the Media Exposure Scale, and loneliness using the Three-Item Loneliness Scale (T-ILS). Sociodemographic characteristics of the participants were also collected. The analysis employed Antonovsky’s sense of coherence (SOC) model, along with univariate and multivariate linear regression analyses, to investigate the factors influencing loneliness.

**Results:**

A total of 4,404 questionnaires were collected, with male participants comprising 53.07% and female participants comprising 46.93% of the sample. The mean age of the participants was 13.60 years, with a standard deviation (SD) of 2.85 years. The mean scores for loneliness, intimate partner violence, perceived stress, and media exposure were 4.70 (SD = 1.70), 7.42 (SD = 3.03), 6.55 (SD = 2.82), and 21.28 (SD = 5.89), respectively. Loneliness was positively correlated with intimate partner violence (*r* = 0.320, *p* < 0.001), perceived stress (*r* = 0.454, *p* < 0.001), and media exposure (*r* = 0.141, *p* < 0.001). A multivariate regression analysis indicated that intimate partner violence (*β* = 0.102, *p* < 0.001), perceived stress (*β* = 0.225, *p* < 0.001), and media exposure (*β* = 0.028, *p* < 0.001) were significant predictors of loneliness.

**Conclusion:**

Intimate partner violence, perceived stress, and media exposure have significant effects on loneliness among Chinese children. Interventions aimed at improving family environments, enhancing resilience to stress, and guiding appropriate media use may effectively reduce loneliness and promote healthy child development.

## Introduction

Loneliness is increasingly recognized as a significant public health concern, intricately linked to both physical and mental well-being (2). It is described as a distinct, unpleasant experience resulting from a lack of sufficient and meaningful social relationships, and it is also understood as the discrepancy between expected and actual social relationships (3). The current status of loneliness among Chinese children and adolescents is characterized by its non-negligible prevalence and far-reaching adverse impacts. A cross-sectional study conducted in China showed that more than 33.9% of participating adolescents experienced loneliness during the COVID-19 pandemic; meanwhile, previous studies have indicated that lonely adolescents are more likely to report poor academic performance, juvenile delinquency, poor general health, depression, and suicidal behaviors. In addition, loneliness experienced in early life may carry over into adulthood, leading to an increased risk of morbidity in later life (4). This issue is particularly acute among school-aged children, with severe loneliness affecting approximately10 to 16% of this population (2). The overall prevalence of loneliness in this population is approximately 20% (5). This distressing emotional condition not only compromises children’s immediate emotional health but also has the potential to adversely impact their social adaptation, academic performance, and long-term mental health (6). Despite the importance of this issue, existing research has predominantly focused on describing the manifestations of loneliness and identifying its risk factors. In contrast, this study adopts a public health perspective that prioritizes “prevention over cure” (7). It systematically investigates strategies to prevent and alleviate loneliness by enhancing individuals’ internal resources and external support systems. This constitutes the central focus of our research. Meanwhile, recent studies have shown that the detection rate of loneliness among middle school students in Shanghai reaches as high as 53.7%, with the detection rates of depression and anxiety being 18.8 and 38.5% (8), respectively. National cross-temporal meta-analyses have also indicated that the level of loneliness among Chinese adolescents exhibited a continuous upward trend from 2001 to 2019, which was particularly prominent in central and western regions and among junior high school students (9). Long-term and high levels of loneliness can severely impair children’s social competence and emotional development, elevate the risk of psychological problems such as anxiety and depression, and exert long-term adverse impacts on their physical and mental health, academic performance, and social adjustment. Nevertheless, authoritative and large-scale epidemiological survey data on this topic remain scarce in China, which underscores the necessity and urgency of conducting the present study.

**Figure 1:**
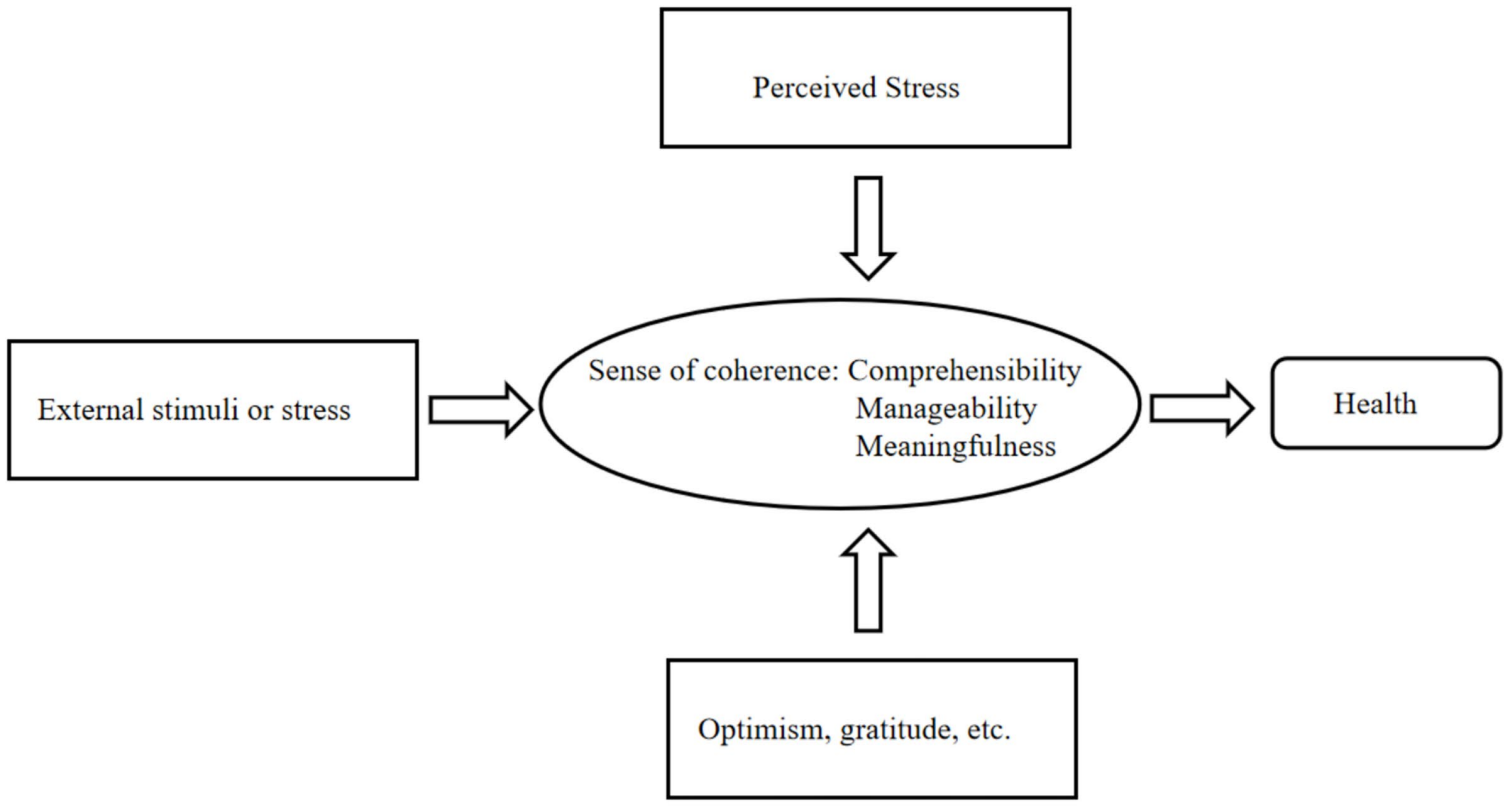
Health-beneficial model.

**Figure 2:**
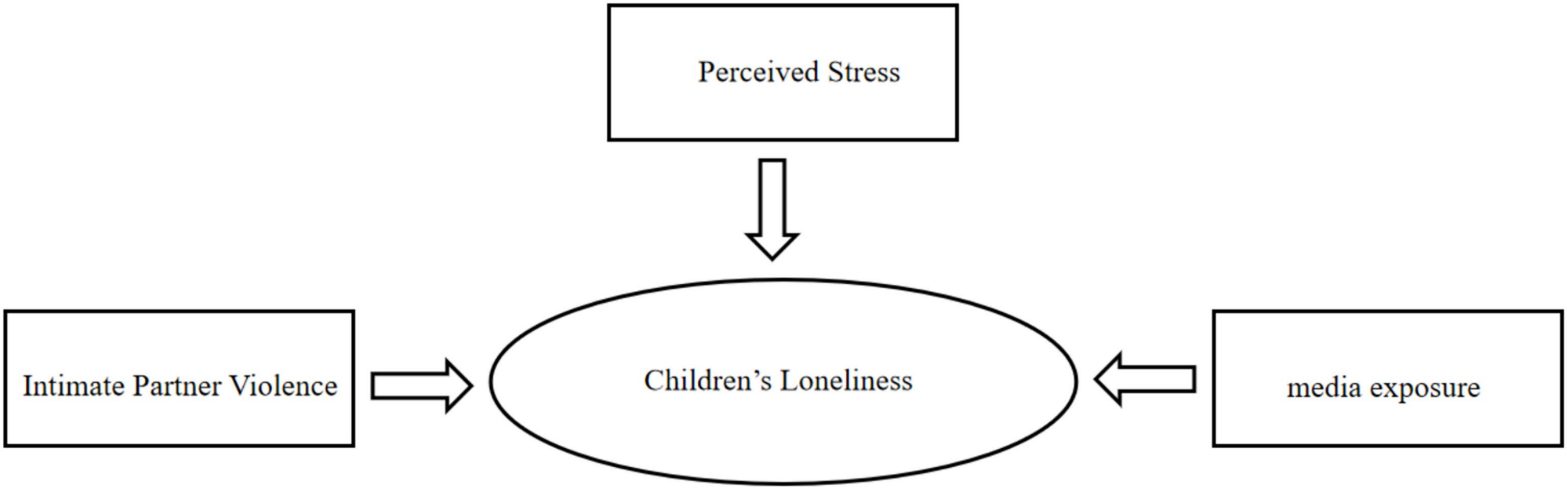
Theoretical framework.

To address the identified research gap, this study employs Antonovsky’s “Salutogenic Model” as its principal theoretical framework. This model facilitates a paradigm shift from the traditional “pathology-oriented” approach to one focused on “health promotion,” thereby redirecting the emphasis from understanding “how stressors cause disease” to identifying “what factors sustain and promote health.” It broadens intervention strategies beyond isolated psychotherapy, encompassing a multi-layered, socioecological integration system that includes individual, social, and ecological dimensions. The model posits that an individual’s capacity to transform external stressors into manageable challenges depends on their sense of coherence (SOC) (10). This SOC is characterized as an individual’s capacity to integrate external demands, internal resources, and social support into a cohesive and meaningful framework. This capacity is regulated by resources available at personal, social, and institutional levels. To clearly present the theoretical logic of this study, the theoretical framework is constructed as follows: Intimate partner violence (IPV) and media exposure serve as external environmental factors that, together with perceived stress as an individual cognitive factor, affect children’s experiences of loneliness. Each variable corresponds clearly to the core components of the theoretical model, making the theoretical structure more complete and explicit. Therefore, this study systematically explores the tri-level mechanism underlying children’s experiences of loneliness, encompassing “personal-social-institutional” factors Figures 1, 2.

At the individual level, perceived stress has been demonstrated as the most consistent proximal predictor of loneliness (11). This concept pertains to an individual’s subjective evaluation of the stress they have encountered over a given period (12). Elevated levels of perceived stress substantially diminish the SOC, thereby exacerbating feelings of loneliness, with interpersonal conflict serving as the primary stressor (13). At the societal level, IPV refers to physical, sexual, and psychological violence, as well as stalking, perpetrated by a current or former intimate partner. Numerous meta-analyses and systematic reviews indicate that it negatively impacts children’s adjustment outcomes, including psychopathological development, academic functioning, and social functioning, and that childhood exposure to IPV further increases the risk of negative long-term consequences in adolescence and adulthood (14). Children frequently become “peripheral victims” or “witnesses” to IPV, and even in the absence of direct abuse, mere exposure to such environments can generate “toxic stress” (15). This exposure undermines their sense of security and trust in peers, thereby intensifying feelings of loneliness (15, 16). Statistics reveal that approximately 1 in 15 children are exposed to IPV annually (16). At the institutional level, media exposure is defined as the behavior of individuals acquiring information and engaging in social interactions through various media channels, such as television, the internet, and social media (1). Excessive internet use among children can potentially hinder typical social and academic development, thereby elevating the risk of loneliness and depression (1).

In conclusion, perceived stress, exposure to IPV, and excessive media consumption may collectively intensify the risk of loneliness among Chinese children by undermining their SOC and deteriorating their social support networks. Utilizing the Salutogenic Model as a framework, this study conducts a systematic examination of the independent, interactive, and cumulative effects of these multi-level factors on children’s experiences of loneliness. The objective is to provide empirical evidence that can inform the development of age-appropriate, cross-system early intervention strategies.

This research utilizes Antonovsky’s Salutogenic Model to conduct an in-depth examination of the impact of intimate partner violence, perceived stress, and media exposure on loneliness among Chinese children. The aim is to establish a scientific basis for comprehending and addressing childhood loneliness. Grounded in the theoretical framework of the Salutogenic Model, we propose the following hypotheses: H1: Intimate partner violence has a positive correlation with children’s loneliness. H2: Perceived stress is positively associated with children’s loneliness. H3: Media exposure is also positively associated with children’s loneliness.

## Methods

### Study design

This research employed survey data to examine loneliness among Chinese children aged 8 to 18 years. Conducted as a nationwide cross-sectional survey, the study sought to elucidate the characteristics of loneliness within this demographic. Data collection occurred from October 2023 to May 2024 and encompassed children’s hospitals in 18 provinces and municipalities throughout China, with the exception of the Hong Kong, Macau, and Taiwan regions. Ethical approval for the study was obtained from the Ethics Committee of Beijing Children’s Hospital, Capital Medical University (Ethics Approval No: 2023-e-015-r).

In this survey, researchers improved the questionnaire through a literature review, expert consultation, and three rounds of pre-surveys; meanwhile, they recruited provincial investigators online and selected qualified personnel to carry out the survey after resume screening, interview review, and online training. During the survey period, the research team collected and answered the problems encountered by each responsible person through the Tencent Meeting every week, conducted logical checks of the questionnaires, and shared the latest results to ensure the smooth progress of the survey.

Provinces and municipalities in mainland China were stratified according to geographic location, level of economic development, and urban–rural distribution to ensure regional representativeness. Within each stratum, children’s hospitals were selected based on service coverage, accessibility, and willingness to cooperate, so as to include institutions representative of local pediatric health services. Researchers established questionnaire stations at the designated children’s hospitals. They recruited participants aged 8 to 18 years, verified their identities, and confirmed eligibility by ensuring adherence to inclusion criteria and the absence of exclusion criteria. Electronic questionnaires were distributed to eligible participants. Informed consent was obtained during the survey process, with researchers either entering the questionnaire ID themselves or providing it to the participants. For respondents who possessed cognitive ability but lacked sufficient functional capacity to complete the questionnaire independently, the investigator conducted a one-on-one interview and completed the questionnaire on their behalf. Quality control measures included the following: (1) allowing only one questionnaire submission per IP address to prevent duplicate responses from the same individual, (2) setting a minimum response time of 240 s, and (3) excluding questionnaires that exhibited logical inconsistencies during validation as invalid responses.

### Participants

The study analyzed a total of 4,404 valid survey responses. The inclusion criteria were as follows: (1) children aged 8 to 18 years; (2) voluntary participation by both children and their guardians, with informed consent forms signed by guardians on behalf of the children; (3) possession of Chinese nationality and permanent residency status, with annual travel duration not exceeding 1 month; and (4) the ability to independently complete the questionnaire or do so with guardian assistance, along with comprehension of each item’s meaning. The exclusion criteria encompassed the following: (1) individuals exhibiting mental confusion or psychotic disorders, (2) those with cognitive impairments, (3) individuals who had previously engaged in similar studies, and (4) individuals unwilling to cooperate. The study was conducted in accordance with ethical standards, received approval, and adhered to the principles of voluntary participation and anonymity, with all participants fully informed of the relevant research protocols.

### Measurements

The IPV scale was developed by the research team to assess children’s exposure to intimate partner violence between their parents. It includes three dimensions—the source of violence, physical violence, and psychological violence, with five items rated on a 5-point Likert scale ranging from “Never” to “Almost always,” scored from 1 to 5. Total scores range from 5 to 25, with higher scores indicating more intimate partner violence. The scale showed strong reliability and validity in this study.

Stress assessment utilized the Perceived Stress Scale-4 (PSS-4) developed by Cohen et al. (17). The scale encompasses the dimensions of loss of control and tension and comprises four items rated on a 5-point scale (0 = Never, 1 = Rarely, 2 = Sometimes, 3 = Often, 4 = Always). Total scores range from 0 to 16, with items 2 and 3 reverse-scored. Higher scores indicate greater perceived stress (18).

The Media Exposure Scale utilized a 5-point Likert scale ranging from 1(Never used) to 5(Almost daily) to assess participants’ frequency of engagement with seven distinct types of media: Newspapers, magazines, radio, television, non-textbook books, personal computers (including tablets), and smartphones. Participants were required to report their usage frequency on a 5-point Likert scale (0 = Never, 1 = Rarely, 2 = Sometimes, 3 = Often, 4 = Always), resulting in a total score range of 0 to 28 points. Higher scores are indicative of increased media exposure frequency.

Loneliness was evaluated using the Three-Item Loneliness Scale (T-ILS) developed by Russell et al., which is designed to provide a swift assessment of an individual’s degree of loneliness. This scale consists of three items, each rated on a 3-point Likert scale (1 = Never, 2 = Sometimes, 3 = Always), resulting in a total score ranging from 3 to 9 points. Elevated total scores are indicative of heightened loneliness. Notably, the second item is reverse-scored. Prior studies have substantiated the T-ILS’s robust validity and reliability (19), and in the current study, the scale demonstrated a Cronbach’s *α* coefficient of 0.890.

### Sampling methods and sample size

Children’s hospitals in 18 provinces and municipalities were selected for this survey. Given the large study population and limited familiarity with its units or elements, probability sampling was strictly adopted to eliminate subjective influence. Equal probability sampling (stratified sampling) was used to make the selected sample more representative and further reduce sampling error. When sampling from children’s hospitals at the individual level, the overall sample size was further reduced, and researchers were able to become familiar with the characteristics of the population. At this stage, non-equal probability sampling (quota sampling) was conducted according to sample attributes, providing a reliable basis for the in-depth analysis of this study.

Data analysis was conducted utilizing SPSS version 27.0. Quantitative data are presented as mean ± standard deviation (SD). Descriptive statistics were employed to examine factors influencing loneliness and participant characteristics. Categorical data are presented as frequencies and percentages. For continuous variables with a normal distribution, descriptive statistics are expressed as the mean and SD, while variables not conforming to a normal distribution are reported as the median and interquartile range (25th and 75th percentiles). Pearson’s correlation coefficients were used to evaluate the relationships between variables. A multivariate linear regression analysis was performed to identify factors influencing loneliness, with loneliness as the dependent variable and intimate partner violence, perceived stress, and media exposure as independent variables, while adjusting for demographic covariates such as age and sex. The significance level was set at *α* = 0.05, with *p*-values less than 0.05 considered statistically significant.

## Results

### Participants’ sociodemographic characteristics

This study included 4,404 participants with a mean age of 13.60 ± 2.85 years. Male individuals represented 53.07% of the sample. Educational attainment was distributed as follows: 32.02% had completed junior high school and 32.02% had completed elementary school. The majority of participants (90.96%) reported no chronic diseases. In terms of residential status, 68.32% had lived in urban areas during the preceding 3 months, while 51.23% held rural household registration. In addition, 68.28% of participants were only children, and 53.1% spent more than 12 h daily with family members. Among cohabitants, 78.50% were not on long-term medication, 83.92% were not medical professionals, and 85.42% did not have private rooms. The results of the univariate analysis are provided in Table 1. Independent samples *t*-tests and analysis of variance indicated statistically significant differences between groups for all demographic variables, with the exception of “cohabitants not being medical professionals” (*p* = 0.125).

**Table 1:** Univariate analysis of loneliness among 4,404 Chinese children with different demographic characteristics (mean ± SD).

Variables	n (%)	Loneliness	T/F	*p*
Sex			4.513	< 0.001
Male children	2,337 (53.07)	4.59 ± 1.69		
Female children	2067 (46.93)	4.82 ± 1.70		
Education level			25.511	< 0.001
Primary school	1,410 (32.02)	4.36 ± 1.50		
Junior high school	1,410 (32.02)	4.64 ± 1.73		
Technical secondary school	612 (13.89)	5. 11 ± 1.85		
Senior high school	852 (19.35)	5.00 ± 1.75		
Junior college	76 (1.73)	5. 16 ± 1.58		
Bachelor’s degree	44 (0.99)	4.89 ± 1.74		
Chronic disease			−10.566	< 0.001
Yes	398 (9.04)	5.55 ± 1.82		
No	4,006 (90.96)	4.61 ± 1.67		
Residential region in the past 3 months			5.237	< 0.001
Rural	1,395 (31.68)	4.90 ± 1.75		
Urban	3,009 (68.32)	4.61 ± 1.67		
Registered residence			5.076	< 0.001
Rural	2,256 (51.23)	4.83 ± 1.73		
Urban	2,148 (48.77)	4.57 ± 1.37		
Only child			−3.105	0.002
Yes	3,007 (68.28)	4.75 ± 1.70		
No	1,397 (31.72)	4.58 ± 1.69		
Family accompaniment time(h/d)			6.140	< 0.001
≤12 h	2,340 (53. 1)	4.85 ± 1.75		
>12 h	2064 (46.9)	4.53 ± 1.63		
Household members on long-term medication			−6.744	< 0.001
Yes	947 (21.50)	5.03 ± 1.73		
No	3,457 (78.50)	4.61 ± 1.68		
Household member as a medical worker			1.535	0.125
Yes	708 (16.08)	4.61 ± 1.72		
No	3,696 (83.92)	4.72 ± 1.70		
Separate rooms at home			5. 144	< 0.001
Yes	642 (14.58)	5.02 ± 1.80		
No	3,762 (85.42)	4.64 ± 1.68		

### Mean, standard deviation, and correlation of variables

The mean scores for the scales measuring loneliness, intimate partner violence, perceived stress, and media exposure were 4.70 ± 1.70, 7.42 ± 3.03, 6.55 ± 2.82, and 21.28 ± 5.89, respectively (refer to Table 2). The correlations among the variables are shown in Table 3. Age demonstrated a significant positive correlation with BMI (*r* = 0.249, *p* < 0.001). Intimate partner violence was positively correlated with BMI (*r* = 0.036, *p* < 0.01), whereas perceived stress showed significant positive correlations with age (*r* = 0.200, *p* < 0.001), BMI (*r* = 0.080, *p* < 0.001), and intimate partner violence (*r* = 0.266, *p* < 0.001). Media exposure was significantly positively correlated with age (*r* = 0.322, *p* < 0.001), BMI (*r* = 0.092, *p* < 0.001), and perceived stress (*r* = 0.057, *p* < 0.001). Loneliness was significantly positively correlated with age (*r* = 0.160, *p* < 0.001), BMI (*r* = 0.054, *p* < 0.001), intimate partner violence (*r* = 0.302, *p* < 0.001), perceived stress (*r* = 0.454, *p* < 0.001), and media exposure (*r* = 0.141, *p* < 0.001).

**Table 2:** Scores of loneliness, intimate partner violence, perceived stress, and media exposure scales among Chinese children (Mean ± SD).

Variables	Scale score
Loneliness	4.70 ± 1.70
Intimate partner violence	7.42 ± 3.03
Perceived stress	6.55 ± 2.82
Media exposure	21.28 ± 5.89

**Table 3:** Correlation analysis of age, BMI, intimate partner violence, perceived stress, media exposure, and loneliness among Chinese children (*n* = 4,404).

Variables	Age	BMI	Intimate partner violence	Perceived stress	Media exposure	Loneliness
Age	1.000					
BMI	0.249**	1.000				
Intimate partner violence	0.029	0.036*	1.000			
Perceived stress	0.200**	0.080**	0.266**	1.000		
Media exposure	0.322**	0.092**	0.014	0.057**	1.000	
Loneliness	0. 160**	0.054**	0.302**	0.454**	0.141**	1.000

### Multivariate logistic regression analysis

The distribution of independent variables is comprehensively presented in Table 4, Table 5 highlights the significant factors influencing the final results, which included sex (*β* = −0.126, *p* = 0.005), presence of chronic disease (*β* = 0.337, *p* < 0.001), duration of family accompaniment (*β* = −0.103, *p* = 0.022), whether cohabitants are on long-term medication (*β* = 0.110, *p* = 0.044), having a private room (*β* = −0.186, *p* = 0.003), intimate partner violence (*β* = 0.102, *p* < 0.001), perceived stress (*β* = 0.225, *p* < 0.001), and media exposure (*β* = 0.028, *p* < 0.001). The overall model fit was significant (*F* = 114.039, *p* < 0.001). The coefficient of determination *R^2^* was 0.267, and the adjusted *R^2^* was 0.264, indicating that the model explained 26.4% of the variance in the dependent variable. Collinearity diagnostics showed that the variance inflation factor (VIF) for all independent variables was less than 5, suggesting no multicollinearity among the variables.

**Table 4:** Independent variable coding table.

Variables	Coding
Age	Original value
Sex	Male = 1, Female = 0
BMI	Original value
Education level	Primary school = 0, Junior high school = 1, Technical secondary school = 2, Senior high school = 3, Junior college = 4, Bachelor’s degree = 5
Chronic disease	Yes = 1, No = 0
Residential region in the past 3 months	Urban = 1, Rural = 0
Registered residence	Urban = 1, Rural = 0
Only child	Yes = 1, No = 0
Family accompaniment time (h/d)	≤12 h = 0, >12 h = 1
Household members on long-term medication	Yes = 1, No = 0
Separate rooms at home	Yes = 1, No = 0
Intimate partner violence	Original value
Perceived stress	Original value
Media exposure	Original value

**Table 5:** Multivariate regression analysis of loneliness in 4404 Chinese children.

Independent variable	B	SE	Beta	*t*	*p*	95% CI
(Constant)	5.173	0.277		18.689	< 0.001	4.630 ~ 5.716
Age	0.001	0.015	0.002	0.077	0.939	−0.029 ~ 0.032
Sex	−0.126	0.045	−0.037	−2.788	0.005	−0.214 ~ −0.037
BMI	−0.001	0.006	−0.003	−0.21	0.834	−0.013 ~ 0.010
Education level	0.063	0.035	0.045	1.79	0.073	−0.006 ~ 0.132
Chronic disease	0.337	0.079	0.057	4.257	< 0.001	0.182 ~ 0.492
Residential region in the past 3 months	−0.013	0.056	−0.004	−0.237	0.812	−0.123 ~ 0.097
Registered residence	−0.034	0.053	−0.01	−0.649	0.516	−0.139 ~ 0.070
Only child	−0.001	0.05	< 0.001	−0.016	0.987	−0.098 ~ 0.097
Family accompaniment time (h/d)	−0.103	0.045	−0.03	−2.291	0.022	−0.191 ~ −0.015
Household members on long-term medication	0.11	0.055	0.027	2.013	0.044	0.003–0.218
Separate rooms at home	−0.186	0.063	−0.039	−2.934	0.003	−0.310 ~ −0.062
Intimate partner violence	0.102	0.008	0.181	13.173	< 0.001	0.087–0.117
Perceived stress	0.225	0.008	0.373	26.827	< 0.001	0.209–0.242
Media exposure	0.028	0.004	0.096	6.968	< 0.001	0.020–0.036
VI*F* value			1.030–4.010			
*R*^2^值			0.264			
*F*-value			114.039			
*p*-value			< 0.001			

## Discussion

The findings of this study provide additional evidence that intimate partner violence, perceived stress, and media exposure have substantial impacts on loneliness among Chinese children. Concurrently, certain demographic variables also demonstrated correlations with loneliness. Regression analysis showed that intimate partner violence (*β* = 0.302, *p* < 0.001), perceived stress (*β* = 0.454, *p* < 0.001), and media exposure (*β* = 0.141, *p* < 0.001) were significantly positively correlated with loneliness.

Within the context of the Health Promotion Model, the development and mitigation of loneliness can be explained through interactions at three levels: Individual, social, and institutional. At the individual level, children who experience elevated perceived stress are prone to negative emotions, which hinder their ability to engage in effective communication and interaction with others, thereby exacerbating their sense of loneliness. At the social level, the family environment is pivotal in the genesis of loneliness. Intimate family interactions—characterized by companionship, understanding, and support—foster a sense of belonging and security in children, thereby diminishing feelings of loneliness. Conversely, exposure to family violence, whether physical or psychological, can inflict psychological trauma, undermine children’s fundamental trust in their family, and lead to withdrawal and isolation in their interpersonal relationships. At the institutional level, media exposure serves as a significant factor. For example, creating supportive media usage environments and fostering inclusive, mutually supportive social cultures can provide a foundation for both individual and societal interventions. Conversely, inadequate institutional safeguards or a social culture overly emphasizing individual independence may indirectly increase the risk of loneliness.

This study found that intimate partner violence significantly and positively influences children’s loneliness, supporting Hypothesis 1 and aligning with previous research findings (20). Domestic violence often disrupts children’s family safety environment and social support systems, leaving them in a state of psychological isolation and helplessness, thereby heightening loneliness (21). Furthermore, intimate partner violence may exert long-term negative effects on children’s mental health, further intensifying their experience of loneliness. Hypothesis 2 was also confirmed, indicating that perceived stress significantly positively influences children’s loneliness. This aligns with existing research findings (22). Elevated levels of perceived stress may cause children to feel uncomfortable in settings, diminishing their social engagement and consequently intensifying their feelings of loneliness. Simultaneously, loneliness may exacerbate individuals’ subjective perception of stress, creating a mutually reinforcing cycle (23). At the institutional level, media exposure exerts a complex influence on children’s experiences of loneliness. Our findings support Hypothesis 3, indicating a positive association between media exposure and loneliness (24). On one hand, media use provides opportunities for information access and social extension, potentially alleviating loneliness. On the other hand, excessive use or dependence on media—particularly when substituting real-world social interactions—may alienate children from reality, thereby increasing loneliness (25). Furthermore, exposure to harmful content may trigger cognitive biases and behavioral issues in children, further intensifying their loneliness. Although social media can facilitate strong identity formation for some users, the lack of sufficient offline interaction and social support may ultimately increase loneliness and related psychological risks over the long term (25).

The findings of this study indicate that IPV directly and positively predicts loneliness while also indirectly intensifying loneliness experiences by elevating perceived stress.

Although media exposure was statistically significantly associated with loneliness in this study (*β* = 0.028), its effect size was substantially smaller than that of other predictors. This finding highlights the marked type-specific specificity of media exposure on loneliness, as a generalized media exposure index fails to distinguish the divergent effects of different media types. Previous studies have demonstrated that social media acts as the core predictor of loneliness, exerting greater effects through mechanisms such as social comparison and low-quality online interaction and even triggering a bidirectional cycle of loneliness and excessive social media use (26, 27). In contrast, educational media, characterized by task-oriented usage, has a negligible direct impact on loneliness, with its weak association attributed primarily to contextual factors (e.g., superficial interaction) rather than the media itself (28, 29). The small overall effect size observed in this study may therefore stem from the offsetting effects of these two media types. A key limitation of the present study is the lack of media type stratification; future subgroup analyses will identify the dominant media type driving loneliness. Moreover, future studies are warranted to investigate the associations and underlying mechanisms among children and adolescents aged 8–18 years, given their critical neurodevelopmental stage and distinct patterns of media use. For Chinese populations, targeted interventions may include reducing passive social media use, integrating online and offline social interactions, optimizing emotional interaction design in domestic educational media, and delivering loneliness-related education via government social media platforms, thereby enhancing the translational value of the findings.

Within the context of Chinese society’s strong emphasis on educational achievement, a positive family environment is undeniably vital for the physical and mental well-being of children (30). The present study was conducted within the Chinese sociocultural context. Unique institutional factors in China include the hukou (household registration) system, which categorizes individuals as urban or rural residents and shapes their access to public services and resources. In addition, urban–rural disparities reflect deep-seated economic and social inequalities between urban and rural regions. Drawing on these findings, we propose a multi-tiered, comprehensive intervention strategy. At the family level, we advocate for the implementation of parenting education programs designed to cultivate harmonious and safe home environments, with a firm commitment to eradicating all forms of domestic violence. At the school and community level, mental health education should be integrated into curricula to enhance children’s stress management and emotional regulation skills, and diverse group activities should be organized to promote offline peer interaction and social connections (31). At the policy and media level, child-friendly online protection policies should be developed and enforced, children should be guided toward healthy media usage habits, and a balanced approach to content consumption and creative engagement should be encouraged.

Although this study demonstrates a commendable level of scientific rigor and representativeness, particularly in terms of sample size and research methodology, there are several limitations. First, the cross-sectional design utilized in this study, although effective in identifying correlations between variables, is insufficient for establishing causal relationships. Future research should consider employing a longitudinal design to provide a more comprehensive understanding of how these influencing factors develop over time and affect children’s experiences of loneliness. Second, as participants were recruited from a pediatric hospital, the study sample may not fully represent the general population. Future research should acknowledge and explicitly discuss the potential selection bias that may arise from children’s health status and family background. Third, this study used univariate and multivariate regression analyses to screen variables and verify their independent predictive effects. In the follow-up, we will use mediation effect analysis, structural equation modeling (SEM), and other methods to further verify the mechanism of action of the theoretical model.

## Conclusion

This cross-sectional study examined the effects of intimate partner violence, perceived stress, and media exposure on loneliness among Chinese children. The findings revealed significant correlations between these factors and childhood loneliness. Intimate partner violence disrupts family environments, perceived stress reduces social interactions, and increased media exposure may amplify individuals’ feelings of loneliness. Utilizing the Health Promotion Model as a framework, this study advocates for a prevention-oriented, multi-level, comprehensive intervention strategy that addresses individual, family, societal, and institutional dimensions, highlighting the necessity of cross-level collaboration. Future research should further evaluate intervention effectiveness, extend to diverse cultural and regional contexts, and validate intervention measures using experimental and longitudinal designs. Such efforts will provide theoretical support and practical evidence for promoting children’s mental health and social adaptation.

## Data Availability

The original contributions presented in the study are included in the article/supplementary material, further inquiries can be directed to the corresponding author.
